# The Present State of Lunacy in England and Wales

**Published:** 1857-10-01

**Authors:** 


### THE PRESENT STATE OF LUNACY IN ENGLAND AND WALES.

The eleventh very able Report recently issued by the Commissioners of Lunacy, presents great variety of interesting and valuable information on the present state and statistics of insanity throughout England and Wales; and cannot fail of being perused with the greatest interest by the psychologist especially, the philanthropist, political economist, and by our readers generally.

This Report brings before us much matter for reflection, both in a moral and social aspect; canvassing on a large and comprehensive scale, yet with clearness and precision, questions and facts of gravest character and importance bearing upon that "most dread affliction," insanity—a malady of already fearfully wide extension, and unquestionably still on the increase.

But were the suggestions and plans of the Commissioners really adopted and carried out, we are sanguine enough to believe that this "direful affliction" might be materially abridged in its duration, and more successfully treated generally. We allude here especially to that large and unfortunate class—"the pauper lunatics"—who are now being gradually brought within the pale of a higher humanity. Yet much remains to be done in "ways and means," before the desideratum of systematic and scientific management can be realized, and we can only endorse our sincere wish, that the Commissioners of Lunacy were invested with even more ample and plenary powers for this purpose than they at present possess.

It is shown in the present Report, that the number of the insane domiciled in asylums, hospitals, workhouses, and licensed houses, on the 1st January, 1857, amounted to 21,344 individuals of both sexes, viz., 10,084 males, and 11,260 females: 16,657 being pauper lunatics, and the remainder, 4687, private patients.

SUMMARY.

| | Private. | | | Pauper. | | | Total Males. | Total Females. | Total Lunatics. |
|---|---|---|---|---|---|---|---|---|---|
| | M. | F. | Total. | M. | F. | Total. | | | |
| Asylums . . . . | 119 | 94 | 213 | 6409 | 7687 | 14,096 | 6,528 | 7,781 | 14,309 |
| Hospitals . . . . | 812 | 744 | 1556 | 95 | 80 | 175 | 907 | 824 | 1,731 |
| Metropolitan Licensed Houses . . . . | 657 | 621 | 1278 | 471 | 828 | 1,299 | 1,128 | 1,449 | 2,577 |
| Provincial Licensed Houses . . . . | 787 | 724 | 1511 | 605 | 482 | 1,087 | 1,392 | 1,206 | 2,598 |
| | 2375 | 2183 | 4558 | 7580 | 9077 | 16,657 | 9,955 | 11,260 | 21,215 |
| Royal Naval Hospital | 129 | ... | 129 | ... | ... | ... | 129 | ... | 129 |
| | 2504 | 2183 | 4687 | 7580 | 9077 | 16,657 | 10,084 | 11,260 | 21,344 |

| | Found Lunatic by Inquisition. | | | Criminals. | | | Chargeable to Counties or Boroughs. | | |
|---|---|---|---|---|---|---|---|---|---|
| | M. | F. | Total. | M. | F. | Total. | M. | F. | Total. |
| Asylums . . . . | 5 | 1 | 6 | 197 | 63 | 260 | 534 | 586 | 1120 |
| Hospitals . . . . | 22 | 15 | 37 | 88 | 20 | 108 | 1 | – | 1 |
| Metropolitan Licensed Houses . . . . | 65 | 45 | 110 | 20 | 13 | 33 | 35 | 69 | 104 |
| Provincial Licensed Houses . . . . | 78 | 45 | 123 | 151 | 29 | 180 | 34 | 14 | 48 |
| | 170 | 106 | 276 | 456 | 125 | 581 | 604 | 669 | 1273 |

At the same date that 1 person in every 701 of the population of England and Wales was of unsound mind—a considerable increase in five years, as will be seen by comparison of the following years:—

In 1852 the ratio was 1 in 847
In 1854 „ 1 — 762
In 1857 „ 1 — 701

The last five years,—from 1852 to 1857,—exhibits an increase of 3,932 private and pauper lunatics, according to the Report of the Commissioners of Lunacy; and that of the Poor-law Board is still higher,—proving a decided increase of insanity beyond that of the increase of population. Of the entire number of lunatics, 3,227 only were deemed curable, and 17,984 incurable, or about 1 in every 6 persons—a very sad and melancholy calculate proportion.

For the maintenance and clothing of the pauper lunatic class alone, taking the rate stated in the Commissioners' Report, at 6s. 8d. each person weekly, requires an outlay of 266,270*l.* 6s. 8d. annually—truly a large draught on the public purse.

In praise of the ability, zeal, and searching investigations of the Commissioners, too much cannot be said; many, very many abuses have been exposed and rectified; but we repeat, much "*hard work*" remains before them in their arduous duties, before "establishments for the insane" have attained that *working* excellence of arrangement and management of which we believe them capable.

As regards establishments for the reception of pauper lunatics—the class to which we shall now principally confine our observations—the Commissioners do not hesitate in expressing their convictions, and give largely the preference to well-conducted middle-sized asylums, over workhouses, hospitals, or licensed houses; considering the last-named establishments as the worst and most objectionable. In this judgment we fully agree.

On the subject of asylums, the Commissioners report a decided advance made both in county and borough asylums during the past year; still, they have to speak with strong regret and disappointment of the continued avoidance, by large numbers of the boroughs and cities, of the provisions of the Legislature in this matter, and of duties imperfectly discharged by county authorities also.

The Commissioners complain of want of accommodation generally, even for present purposes being sadly deficient; how much more so must they soon become, taking into consideration the increasing ratio of population, and the increasing ratio of insanity to population.

The amount of space allotted to the thirty-three county and four borough asylums, which comprise all the public accommodation yet provided for the lunatic poor, suffices only for the reception of 15,600 patients at the present time, and contain at present 14,309; that many of these establishments have attained their full limit of size, available space remaining for little more than 1300 additional inmates; and during last year, taking the aggregate of ten of these asylums only, nearly 1000 patients were unable to find admission for want of necessary room. And if we couple this with another fact (Appendix to Report, E), it appears that while space has had to be found in licensed houses for nearly 2000 pauper lunatics, for whom there is no available accommodation in county asylums or hospitals, the number of additional lunatics and idiots detained in workhouses, or with friends, amounted, on the 1st January, 1857, to no less than 12,297. The obvious conclusion is, that what was found necessary at Colney Hatch and Hanwell must soon become a general requirement, and that no temporary expedients will satisfy a want so steadily increasing.

To meet these requirements, the Commissioners suggest the building of *additional*, differently constructed, more simple and economical edifices, rather than enlargement of existing asylums generally; and illustrate their position by reference to the already overgrown state of Hanwell and Colney Hatch, which institutions are still undergoing, or about to undergo, increased dimensions: and we entirely concur in the opinion of the Commissioners, that it would have been in every way preferable and wiser to have erected a third asylum of a more simple and less expensive kind. Moreover, beyond a certain size, asylums are objectionable; they forfeit the advantage—which nothing can replace, whether in general management or the treatment of disease—of individual and responsible supervision; few aids being so important in the cure or alleviation of insanity as those derivable from vigilant observation of individual peculiarities: but where the patients are so numerous that no medical officer can bring them within range of his personal examination and judgment, such opportunities are altogether lost; and amid the workings of a great machine, the physician and patient lose alike their individuality. Besides, the more extended the establishment, the more abridged become its means of cure; and this should be the first object of any and every asylum, there being a danger—as in the examples of Hanwell and Colney Hatch—instead of being hospitals for the treatment and relief of insanity, according to their original intention, of becoming permanent places of refuge for too large a proportion of such cases, in which the chances of relief are few, to the exclusion of cases of more recent standing, which, by timely medical care therein, might never have contributed, as they now so largely do, to the permanent burdens on the ratepayers; besides limiting space, and thereby preventing sufficient proper and healthful opportunities of exercise and employment, indispensable to any due treatment of the insane.

To workhouses as receptacles for the insane, their disadvantages greatly outweigh any small benefit that may accrue from such establishments, in the opinion of the Commissioners. The result is, that detention in workhouses not only deteriorates the more harmless and imbecile cases, to which originally they are not unsuited, but has the tendency to render chronic and permanent such as might have yielded to early care; the one class, no longer associated with the other inmates, but congregated in separate wards, rapidly degenerate into a condition requiring all the attendance and treatment to be obtained only in a well-regulated asylum; and the others, presenting originally every chance of recovery, but finding none of its appliances and means, rapidly sink into that almost hopeless state which leaves them for life a burden on their parishes. Nor can a remedy be suggested as long as this workhouse system continues. The attendants are generally pauper inmates, totally unfitted for the charge; the wards gloomy, unprovided with means of occupation, exercise, or amusement; and the diet—above all essential to the unhappy objects of mental disease—rarely in any case exceeds that allowed to healthy and able-bodied inmates.

We might add many more and serious objections, did space permit, to workhouses as receptacles for the insane. We must dismiss very summarily the third division of our analysis—viz., that which relates to licensed houses as habitations for pauper lunatics; and we shall quote the opinions of the Commissioners of Lunacy, as expressed at pages 17 and 18 of their present Report:—

"That existing licensed houses in any adequate respect supply the want to which we have been directing attention—even where the means are large and ample—it is impossible to admit. The accommodation is necessarily of an inferior order; and it is never possible entirely to suppress a question as to the disinterestedness of those with whom the duty rests of receiving, treating, and detaining the inmates. So long as the patient is in a public asylum, no motive exists on the score of economy for depriving him of any comforts which his case requires; but in private institutions it is otherwise, where the same advantages do not exist, and where the difficulty of enforcing recommendations for his benefit can only be appreciated by those upon whom the duty of inspecting these establishments is imposed. The Commissioners in their Report, pages 18, 19, 20, give ample proof of these positions."

The inferences to be drawn from the foregoing data may be summed up as follows:—

1st. That insanity is on the increase.

2nd. That the best means of cure or alleviation of mental disease, especially as bearing upon pauper lunacy, are, well-conducted, airy, and middle-sized asylums.

3rd. That all other *existing* establishments whatever, for the "poor lunatic," are unfit, ill-arranged, and badly conducted.

